# Prenatal Exposure to Air Pollutants and Attentional Deficit Hyperactivity Disorder Development in Children: A Systematic Review

**DOI:** 10.3390/ijerph20085443

**Published:** 2023-04-07

**Authors:** Sharanpreet Kaur, Paula Morales-Hidalgo, Victoria Arija, Josefa Canals

**Affiliations:** 1 Nutrition and Mental Health (NUTRISAM) Research Group, Universitat Rovira i Virgili, 43201 Reus, Spain; sharanpreet.kaur@urv.cat (S.K.); paula.morales@urv.cat (P.M.-H.); victoria.arija@urv.cat (V.A.); 2 Research Center for Behavior Assessment (CRAMC), Department of Psychology, Universitat Rovira i Virgili, 43007 Tarragona, Spain; 3 Department of Psychology and Education Studies, Universitat Oberta de Catalunya (UOC), 08018 Barcelona, Spain; 4 University Research Institute on Sustainablility, Climate Change and Energy Transition (IU-RESCAT) Universitat Rovira i Virgili, 43003 Tarragona, Spain; 5 Department of Basic Medical Sciences, Universitat Rovira i Virgili, 43002 Reus, Spain

**Keywords:** ADHD, hyperactivity, air pollutants, prenatal, children, pregnancy

## Abstract

Up to 9.5% of the world’s population is diagnosed with attention deficit/hyperactivity disorder (ADHD), making it one of the most common childhood disorders. Air pollutants could be considered an environmental risk condition for ADHD, but few studies have specifically investigated the effect of prenatal exposure. The current paper reviews the studies conducted on the association between prenatal air pollutants (PM, NO_x_, SO_2_, O_3_, CO and PAH) and ADHD development in children. From the 890 studies searched through PubMed, Google Scholar, Scopus, and Web of Science, 15 cohort studies met the inclusion criteria. NOS and WHO guidelines were used for quality and risk of bias assessment. The accumulative sample was 589,400 of children aged 3–15 years. Most studies reported an association between ADHD symptoms and prenatal PAH and PM exposure. Data available on NO_2_ and SO_2_ were inconsistent, whereas the effect of CO/O_3_ is barely investigated. We observed heterogeneity through an odd ratio forest plot, and discrepancies in methodologies across the studies. Eight of the fifteen studies were judged to be of moderate risk of bias in the outcome measurement. In a nutshell, future studies should aim to minimize heterogeneity and reduce bias by ensuring a more representative sample, standardizing exposure and outcome assessments.

## 1. Introduction

According to the World Health Organization (WHO) report, almost 99% of the world’s population inhales toxic air that does not meet the thresholds suggested by their guidelines [1], and it is ranked as the fifth most major factor for causing disease globally [2]. The major public health damaging air pollutants are particulate matter (PM), nitrogen dioxide (NO_2_), ozone (O_3_), sulphur dioxide (SO_2_), and carbon monoxide (CO) [1]. Exposure to high concentrations of air pollutants not only damages physical health but also affects neurodevelopment early in life. The prenatal period is a particularly vulnerable developmental window to the effects of air pollution on the brain [3]. The risk of neuroinflammation, damage to neurons and microglia activation increases when exposed to air pollution during pregnancy [4], hence increasing behavioural and cognitive problems in children [5,6,7].

Among neurodevelopmental conditions, attentional deficit hyperactivity disorder (ADHD) is one the most common childhood disorders [8], whose average prevalence is between 3.4% and 9.5% [9,10,11]. ADHD is defined as a persistent pattern of inattention and/or hyperactivity–impulsivity that interfere with functioning or development [12]. It implies a significant personal, familial, and economic impact on society and the health system during childhood and across one’s lifespan [13,14]. Although ADHD is a highly heritable disorder, environmental factors account for between 10% and 40% of variance [15]. Prenatal conditions, such as premature birth [16], maternal pre-pregnancy obesity and overweight, pre-eclampsia, hypertension, acetaminophen exposure, and smoking during pregnancy, have been also strongly associated with ADHD [17,18]. Along with these, ADHD has also been linked to environmental variables like air pollution which could lead to epigenetic changes in genes involved in ADHD [19].

ADHD has been associated with air pollutant exposure to PM [20] and NO_2_ [21] in early childhood. Even the short-term exposure to PM, NO_2_, and SO_2_ may aggravate ADHD symptoms in children and adolescents with ADHD [22]. Prenatal and early exposure to PM_2.5_ reduces the cubic centimetre volume of children’s brains and reduces cognitive abilities, hence increases behavioural problems [23,24]. Additionally, the exposure to Polycyclic aromatic hydrocarbon (PAH), a component of PM_2.5_, during pregnancy has been found to reduce white matter on the left side of the brain and intelligence quotient (IQ), as well as increase the development ADHD symptoms in children [25]. The exposure to PM, NO_2_, and O_3_ during pregnancy is, in addition, related to several ADHD risk factors, such as neonatal respiratory difficulties [26], preterm birth, and early term birth [16,26,27]. The exposure to naturally occurring toxic metals during the gestation and postnatal periods, such as lead (Pb), arsenic (As), cadmium (Cd), copper (Cu), and mercury (Hg), have been found to be associated with ADHD [28,29].

Even though exposure to air pollutants in prenatal stages or early life affects neurodevelopment and can lead to serious lifelong impairments [30], its significance has been under-recognised and often gone unnoticed making it a “silent killer” [31]. This may be due to the difficulty in measuring these effects and the presence of unconverging evidence of such association, although only few studies have found no association between air pollutant exposure during pregnancy and the development of ADHD in children [20,32]. Differences in methodological approaches and inconsistency in prenatal air pollutants exposure concerning ADHD findings point towards the need for a thorough investigation of the existing literature, and therefore, some studies have attempted to approach this topic from a meta-analytical or systematic review approach.

Previous systematic reviews and meta-analyses have investigated the relationship between ADHD and air pollutants with a primary focus on postnatal exposure [28,33]. Donzelli et al. (2020) [33] focused on PM exposure during childhood, and Daneshparvar et al. (2016) [28] focused on lead (Pb) exposure in childhood. The systematic reviews by Aghaei et al. (2019) [19] and Zhang et al. (2020) [34] analysed the association of ADHD and both prenatal and postnatal exposure to ambient gaseous up to April, 2018, and July, 2019, respectively, but only three prenatal studies were finally included. Only the systematic review from Volk et al. (2021) [35], which included studies until July 2017, focused exclusively on prenatal air pollutants and neurodevelopmental disorders. Results from these reviews suggest a diverse relation between ADHD and air pollutants; however, limited and heterogenous data are available on the effect of that exposure during the pregnancy stage and the development of ADHD in children.

Having inadequate data on prenatal air pollution exposure and ADHD demands further investigation of the studies that have explored this topic. Therefore, this systematic review aims to update new published studies and increase the information on the effect of prenatal exposure to air pollutants (such as PM, NO_2_, SO_2_, O_3_, CO, and PAH) in ADHD diagnosis and symptoms.

## 2. Materials and Methods

The present systematic review followed the preferred reporting items for systematic reviews and meta-analysis (PRISMA) updated guidelines from 2020 [36]. The focus of the review is to analyse those studies investigating the association between air pollutant prenatal exposure (PM, NO_2_, SO_2_, O_3_, CO and PAH) and ADHD in children. For this purpose, we included studies in relation to the objective of the study from the last 10 years (2012–2022). We excluded studies investigating health outcomes related with neuropsychological functions or neurophysiological data in ADHD. The literature searches were conducted independently by three investigators through Google Scholar, MEDLINE (through PubMed), Scopus and Web of Science since September 2022. The following word/search strategy was used: (“air pollution” OR pollut* OR “environment pollution”) AND (ADHD OR “attention deficit” OR “hyperactivity disorder” OR “hyperactivity”) AND (preg* OR pren*).

We also searched for articles adding each pollutant by replacing the term (“air pollution” OR pollut* OR “environment pollution”) with words such as: (“particulate matter”); (“nitrogen oxide”); (“sulphur dioxide”); (“ozone”); (“carbon monoxide”) and (“polycyclic aromatic hydrocarbon” OR “PAH”).

### 2.1. Selection Process

All the articles were collected from the search results in the listed databases using references and the bibliographic software Zotero. Duplicate articles were merged. We deleted articles that were not in English, Spanish, French or Italian, and excluded previous systematic reviews from the search. Books, book sections, conference papers, and theses were also removed in the identification process. The selection from the remaining articles was caried out by screening the titles and abstracts following the inclusion and exclusion criteria (Supplementary Table S1). This process was also caried out independently by three reviewers. As inclusion criteria, only epidemiological studies exploring the association between air pollutants (PM, NO_2_, SO_2_, O_3_, CO, and PAH) during pregnancy and its effect on ADHD in children were selected for the systematic review. The exclusion criteria consisted of studies focusing on adults, in vitro and animal effects, other environmental pollutants (tobacco smoke, heavy metals, pesticides, and bisphenol A), other neurodevelopmental disorders (e.g., autism), and controlled trials studies. The screening process was performed by three independent investigators (SK, JC, and PM). The selected articles underwent “Newcastle-Ottawa scale” (NOS) quality assessment and WHO risk of bias assessment in parallel by two investigators (SK and JC), who discussed the results for a mutual conclusion.

### 2.2. Data Extraction

Data from the selected articles were extracted by creating a table listing authors, year of publication, data collection country, study design, participants, air pollutants exposure time, pollutants measured, ADHD diagnosis and symptoms, covariates, and results.

### 2.3. Quality and Risk of Bias Assessment

The 8-item Newcastle–Ottawa scale (NOS) [37] was used to rate the quality of the cohort studies included. The items from the NOS scale are grouped in three different sections: selection (with rates ranging from 0 to 4 stars), comparability (from 0 to 2 stars), and exposure (from 0 to 3 stars). For the selection and exposure section, one star can be given for each item, whereas a maximum of 2 stars can be given for an item in the comparability section. Therefore, the rating for a study cannot be greater than 9 stars in total. The star rating from NOS indicates the quality of the study: 9–8 (very good), 7–6 (good), 5–4 (satisfactory), and 3–0 (unsatisfactory). Using the NOS also allowed us to perform the conversion to meet the Agency for Healthcare Research and Quality (AHRQ) standards as follows: good quality (“3 or 4 stars in selection domain” AND “1 or 2 stars in comparability domain” AND “2 or 3 stars in outcome/exposure domain”), fair quality (“2 stars in selection domain” AND “1 or 2 stars in comparability domain” AND “2 or 3 stars in outcome/exposure domain”), and poor quality (“0 or 1 star in selection domain” OR “0 stars in comparability domain” OR “0 or 1 star in outcome/exposure domain”).

Furthermore, the risk of bias assessment was conducted following the WHO risk of bias assessment instrument for systematic reviews informing WHO global air quality guidelines [38]. Each study was scored on six different domains: confounding, selection bias, exposure assessment, outcome assessment, missing data, and selective reporting. The overall score on each domain was scored as follows: low, moderate or high risk. This can be found in Supplementary Table S2.

## 3. Results

### 3.1. Study Selection

**Figure 1:**
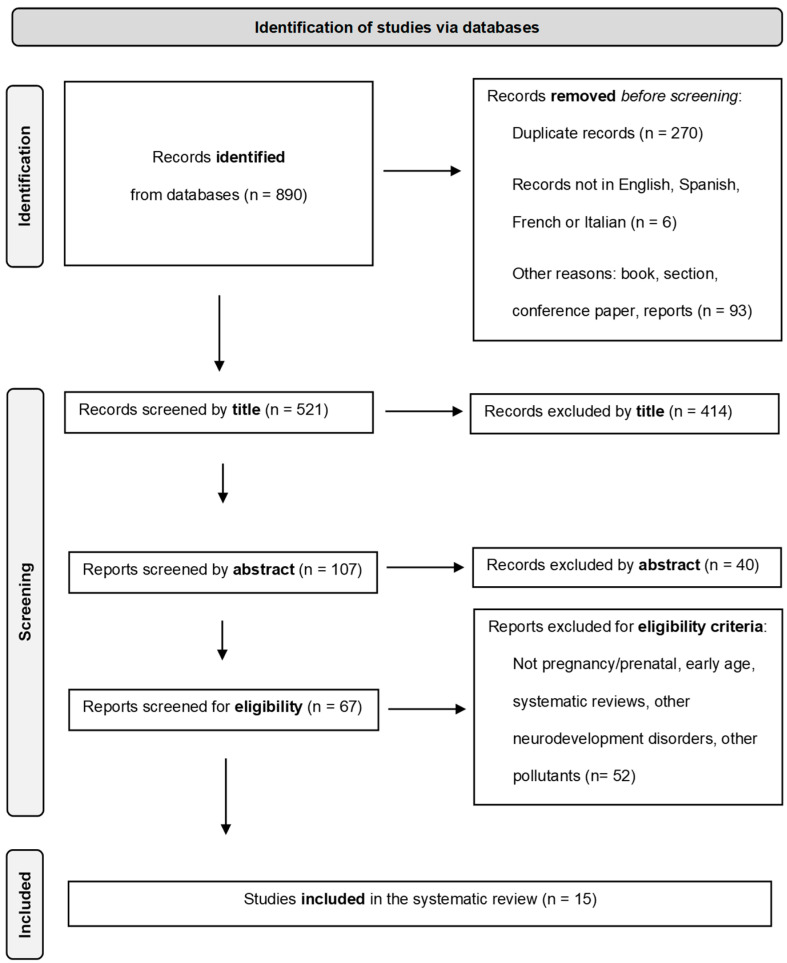
PRISMA-2020-based flow diagram of the selection process in the systematic review.

A total of 890 studies were identified from the four different databases. Out of these, duplicates were removed, and studies were removed for other reasons as well, such as language, books, book sections, reports, and conference papers. The remaining 521 records were screened based on the titles, abstracts, and eligibility based on the inclusion and exclusion criteria. In the screening process, 506 articles were excluded, reducing the number of articles included in the systematic review to 15. A detailed PRISMA flow diagram can be seen in Figure 1.

### 3.2. Studies Characteristics

**Figure 2:**
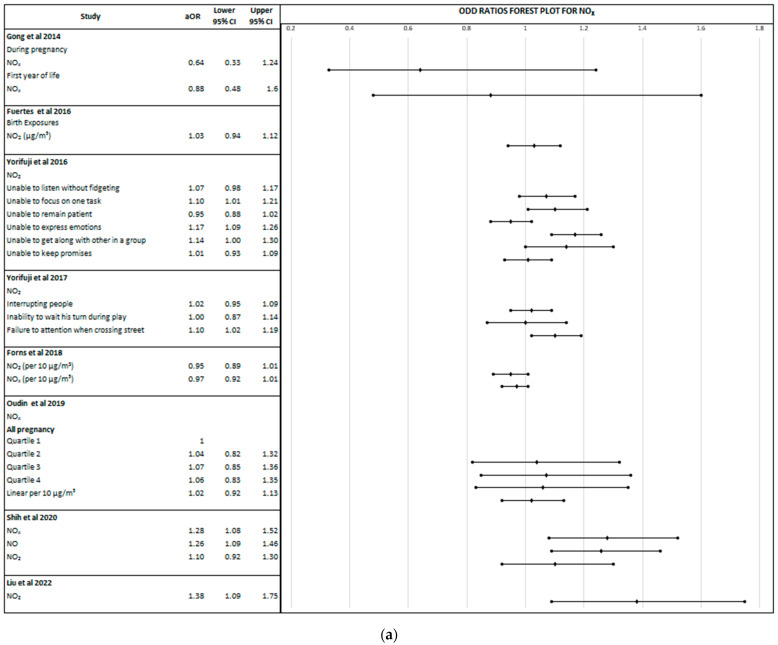

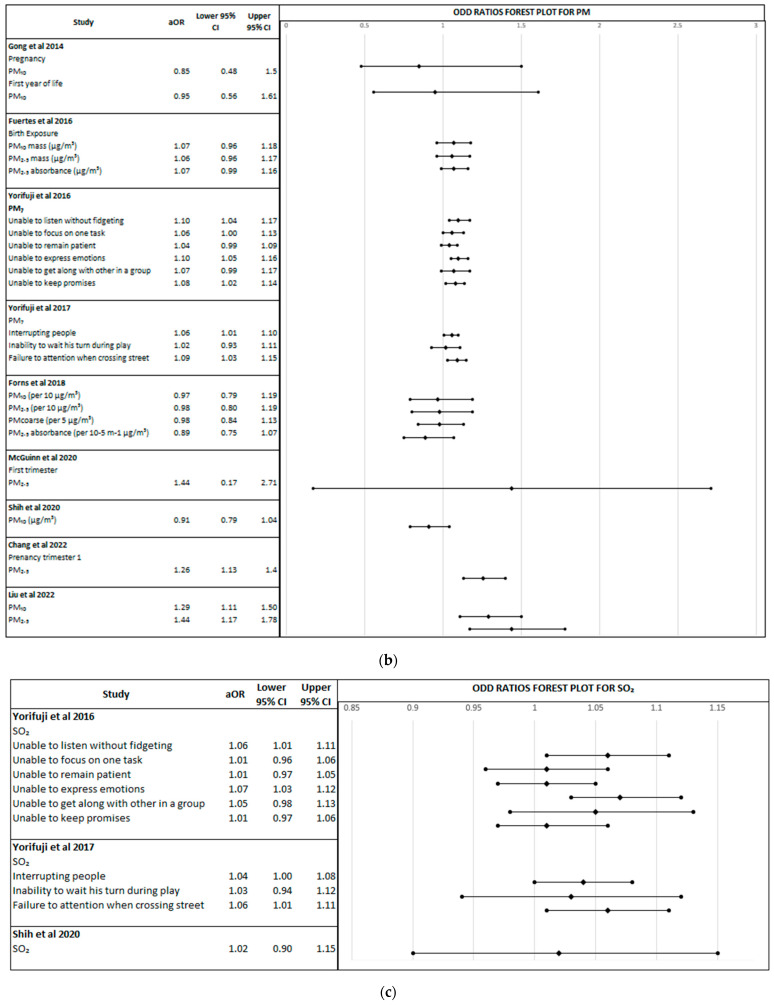

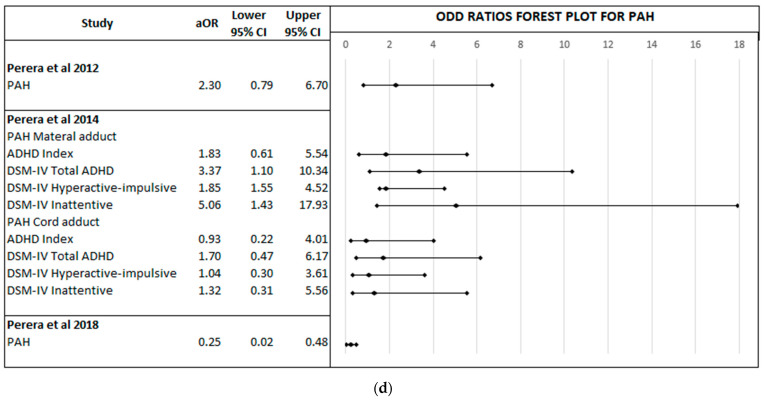
Odd ratios forest plot for each pollutant. (**a**) Odd Ratio Forest Plot of Association between NOx Exposure and ADHD [52,54,55,56,57,59,62,65]; (**b**) Odd Ratio forest plot of association between PM exposure and ADHD [52,54,55,56,57,61,62,64,65]; (**c**) Odd Ratio forest plot of association between SO_2_ exposure and ADHD [55,56,62]; (**d**) Odd Ratio forest plot of association between PAH exposure and ADHD [51,53,58].

Table 1 shows the characteristics of the selected studies. Samples were collected from 12 different countries: Denmark, the Netherlands, Germany, Italy, Spain, Sweden, France, USA, Taiwan, China, Mexico, and Japan. The total number of participants was 589,400, with the range of participants per study being between 253 and 425,736. The effect of air pollutant exposure was assessed in children from 3 to 15 years old. Most of the studies, 12 (80%), analysed ADHD using tools/questionnaires, such as Autism-Tics, ADHD and other Comorbidities (A-TAC) [39], the Strengths and Difficulties Questionnaire (SDQ) [40,41], ADHD-DSM-IV list [42], ADHD Rating scale-IV [43], Conners Parent Rating Scale Revised [44,45], Children Behaviour Check List (CBCL) for ages 1 ½–5 [46], for ages 6–18 [46], and for ages 4–18, Japanese edition [47], Behaviour Assessment System for Children [48] and ad hoc national surveys. Only 3 (20%) studies were based on clinical ADHD diagnoses, using international criteria, such as ICD-10 [49] or ICD-9-CM [50]. Furthermore, the observation of air pollutants studied in the papers also varied as follows: 10 (66.6%) studies were on on particulate matter (PM_10_, PM_2.5 mass_, PM_2.5 absorbance_ and PM_coarse_); 8 (53.3%) studies were on NO_x_/NO/NO_2_; 5 (33.3%) studies were on PAH and 3 (20%) studies on SO_2_, whereas only 1 study (6.67%) studied O_3_ and CO association with ADHD in children. Prenatal toxic exposure measurement includes several methods: general traffic air monitoring using Gaussian dispersion, land-use regression model, satellite-based estimation model, personal air monitoring, as well as maternal and cord blood samples. The studies considered different covariates: (1) From the child: gender, birth weight, age at assessment, child ethnicity, age at follow up, child second-hand smoke exposure, child anxiety/depression, time spent outside, time spent in front of the screen, and non-verbal intelligence; (2) From the mother: environmental tobacco smoke exposure (ETS), gestational age, maternal IQ, maternal education, ethnicity, maternal smoking during pregnancy, maternal marital status, maternal ADHD, mother height, and pre-pregnancy weight; (3) From the family or environment: single parent status, parental psychopathology, parity, family income and family size, prenatal demoralization, heating season, quality of a child’s home environment (HOME), neighbourhood deprivation index at a child’s birth, type of living area, material hardship, parental birth country, mosquito coils, and home renovations. The odd ratio in association with NO_x_, PM, PAH, and SO_2_ exposure and ADHD in children provided by the studies can be visualized in the forest plots, which suggest heterogeneity between them (Figure 2).

### 3.3. Studies Results

#### 3.3.1. Nitrogen Oxides

Four out of the eight studies found an association between prenatal NO_2_ and ADHD onset. Shih et al. (2020) [62] found that NO_x_ was related to hyperactivity symptoms, but further analysis suggested a stronger relation between ADHD and NO than with NO_2_ adjusted odd ratios (aOR) [NO: aOR = 1.26, 95% CI (1.09–1.46)]. Yorifuji et al. (2016, 2017) [55,56] also found NO_2_ exposure during pregnancy to be strongly correlated with ADHD symptoms, such as interrupting people [aOR = 1.02, 95% CI (0.95, 1.09)], inability to wait his/her turn during play [aOR = 1.00, 95% CI (0.87, 1.14)] or failure to pay attention when crossing the street [aOR = 1.10, 95% CI (1.02, 1.19)]. Liu et al. (2022) [65] found a significant increase in hyperactivity in children when exposed to NO_2_ [OR = 1.04, 95% CI (1.06–1.07)] during the seventh month of pregnancy and up to four months after birth, but a peak was observed at nine months of pregnancy. However, the remaining four studies by Gong et al. (2014) [52], Fuertes et al. (2016) [54], Forns et al. (2018) [57], and Oudin et al. (2019) [59] did not find any significant or clear evidence to support the association between NO_x_ and the risk of developing ADHD.

#### 3.3.2. Particulate Matter

Six out of the ten studies supported the association between PM and ADHD. Fuertes et al. (2016) [54] found PM_2.5_ to be associated with hyperactivity/inattention symptoms, PM_2.5 mass_ at 10 and 15 years of age [OR = 1.12, 95% CI (1.01–1.23)] and [OR = 1.11, 95% CI (1.01–1.22)], respectively, and PM_2.5 absorbance_ at 10 and 15 years of age [OR = 1.14, 95% CI (1.05–1.25)] and [OR = 1.13, 95% CI (1.04–1.23)], respectively. Yorifuji et al. (2016, 2017) [55,56] found the pollutant to be associated with ADHD symptoms, such as interrupting people [aOR = 1.06, 95% CI (1.01, 1.10)], inability to wait his/her turn during play [aOR = 1.02, 95% CI (0.93, 1.11)] or failure to pay attention when crossing the street [aOR = 1.09, 95% CI (1.03, 1.15)].

McGuinn et al. (2020) [61] suggested an association between prenatal first trimester PM_2.5_ exposure and increases in attention problems and hyperactivity, but not a significant one. Chang et al. (2022) [64] found that ADHD was significantly associated with a 10 μg/m^3^ increase in PM_2.5_ during the first trimester [OR = 1.26, 95% CI (1.13–1.40)], and increased at PM_2.5_ over 16 μg/m^3^. Furthermore, Liu et al. (2022) [65] found a significant increase in hyperactivity in children when exposed to PM_10_ [OR = 1.04, 95% CI (1.06–1.07)] and PM_2.5_ [OR = 1.06, 95% CI (1.02–1.10)] during the seventh month of pregnancy and up to four months after birth but the peak is observed at nine months of pregnancy, whereas the remaining four studies by Forns et al. (2018) [57], Gong et al. (2014) [58], Shih et al. (2020) [62], and Peterson et al. (2022) [63] did not find an association between PM and ADHD.

#### 3.3.3. Polycyclic Aromatic Hydrocarbons

Most of the studies (four out of five) found a significant association between PAH and ADHD. The study from Peterson et al. (2022) [63] was the only one indicating no association. Perera et al. (2012, 2014) [51,53] supported that high prenatal PAH exposure was positively associated with emotional and attention symptoms. Perera et al. (2014) [53] also found a significant association of this pollutant with the following CPRS-R subscales: DSM-IV Inattentive [OR = 5.06, 95% (CI 1.43, 17.93)] and DSM-IV ADHD Total [OR = 3.37, 95% CI (1.10, 10.34)]. Furthermore, they found that children with high prenatal PAH exposure developed more ADHD symptoms than those with low prenatal PAH exposure [58]. A similar association was found by Pagliaccio et al. (2020) [60], who also described a stronger positive association between being exposed to prenatal PAH and attention problems on the CBCL subscale.

#### 3.3.4. Sulphur Dioxide

Two out of the four studies found an association between prenatal SO_2_ exposure and ADHD symptoms; however, an elevated exposure effect was observed at the age of 2.5 years for interrupting people [aOR = 1.04, 95% CI (1.00, 1.08)], inability to wait for his/her turn during play [aOR = 1.03, 95% CI (0.94, 1.12)], and failure to pay attention when crossing the street [aOR = 1.06, 95% CI (1.01, 1.11)] [54,55], whereas Shih et al. (2020) [62] and Liu et al. (2022) [65] did not find significant results in relation to SO_2_ and hyperactivity.

#### 3.3.5. Carbon Monoxide and Ozone

Out of all the 15 identified studies, only Liu et al. (2022) [65] studied the relationship between CO and O_3_ and prenatal exposure and hyperactivity; however, they did not observe any significant association between the air pollutants and child hyperactive behaviours.

### 3.4. Risk Bias and Quality Assessment

The selected studies were assessed with NOS for quality and risk bias assessment. The minimum score achieved by the studies was 6, and the maximum was 9. Table 2 shows quality stars given based on NOS conversion to AHRQ; all studies selected for the review are of good quality. Table 3 presents a visual representation of the risk of assessment in the form of a heat map. The complete NOS and WHO risk of bias assessment can be found in Supplementary Table S2.

## 4. Discussion

Several investigations have been conducted on the relationship between air pollutants and ADHD across development, but few have looked at the prenatal stage. Therefore, the current systematic review was conducted on 15 cohort studies from the past decade analysing air pollutant exposure during pregnancy, and later, its effect on the development of ADHD in children. The results from the systematic review suggest that PAH and PM are the most strongly associated with ADHD, and specifically, with symptoms of hyperactivity and developmental delays. For prenatal NO_x_ and SO_2_ exposure, half of the studies suggest an association with ADHD, whereas data available on prenatal O_3_ and CO exposure effects are barely existent. Even though some studies observed the relationship between the two factors, the OR values were small, suggesting a low impact of prenatal air pollutant exposure on ADHD development. The forest plot of the odd ratio in the studies shows great heterogeneity between studies and air pollutants. Furthermore, the risk of bias in the outcome measurement was moderate in eight of the fifteen of the studies.

The selected studies are from different continents (Asia, America, and Europe), as well as from both low and high socioeconomic status (SES) countries; hence, a difference is observed in the association between air pollutants and ADHD according to geographical location. The European Environment Agency (EEA, 2019) [66] highlighted the presence of different toxicity profiles depending on the socioeconomic status (SES) of each country. When compared with high and low SES countries, an increased risk of developing chronic problems, including ADHD, was observed in low SES countries due to high concentrations of air pollutants (Russell et al., 2015) [67]. However, this pattern is not observed in the studies identified in the systematic review. The ADHD relation with PAH was found in studies from USA, PM in Europe (mainly in Germany), China and Mexico, NO_x_ in Taiwan and China, while Japanese studies observed a relationship between PM, NO_x_, and SO_2_. An explanation could be that in high SES countries pollution comes from traffic and buildings with poor infiltration of outdoor pollution such as PM and NO_2_. The chemical reaction happening between air pollutants and indoor surfaces produces harmful gases that can have a deteriorating effect on individuals’ health and cause further issues during pregnancy to the growing foetus via the bloodstream [68,69,70].

Studies in the systematic review measured ADHD symptoms at different ages, ranging from 3 to 15 years of age. More often children are left undiagnosed until they are schoolchildren or teenagers. This happens more with less obvious symptoms, such as inattentive symptoms, in comparison to hyperactive/impulsive symptoms [71]. Furthermore, ADHD is an evolutionary neurodevelopment disorder, where symptoms change with age [72]. Therefore, the fact that ADHD symptoms were analysed at different ages in each study could also be a reason for the contrasting results.

ADHD is considered a hereditary disorder in which genes and prenatal risk factors play fundamental roles in the pathogenesis [73]. Hence, children with a genetic vulnerability to ADHD may show even greater vulnerability if exposed to contaminants. Although the studies in the systematic review considered several different covariates, only Perera et al. (2014 and 2018) [53,58] carefully addressed maternal ADHD as a covariate. Furthermore, second-hand smoking exposure in utero and maternal smoking habits are also crucial confounding variables to consider when studying ADHD [74] and other neurodevelopmental disorders in children [18,75]. Here, only half of the studies considered second-hand smoking exposure or smoking during pregnancy as covariates, and this can yield different results, making it hard to draw conclusions.

Furthermore, studies have been conducted on different sizes of air pollutants particles. For example, the size of PM pollutants was measured differently, such as PM_10_ or less, PM_2.5_, PM_coarse_ and PM_absorbance_. Schraufnagel (2020) [76] suggests that ultrafine particles (less than 0.1 µm), which are present in abundance in the environment, are responsible for causing major health risks. In the identified studies, none of them explored less than 0.1 µm particles effect. Additionally, not all studies analysed the effect of different nitrogen oxides (NO_x_). Most of them analysed the prenatal effect of only nitrogen dioxide (NO_2_) and not nitrogen oxide (NO), which is equally important to assess.

An accurate underlying biological mechanism explanation of how prenatal air pollutants exposure develops ADHD in children remains to be elucidated. Nevertheless, some studies have provided a possible explanation of how prenatal air pollutant exposure can affect foetal neurodevelopment and snowball the chances of ADHD in children. Nevertheless, it has been suggested that during pregnancy, since the mother is directly exposed to air pollutants, the ultrafine particles can flow into the female bloodstream through the respiratory system, and they can also increase the production of systematic oxidative stress and inflammatory markers, causing neurodevelopmental damage to the unborn child [70,77]. Furthermore, some studies have attempted to demonstrate how air pollutants such as PM_2.5_, PAH, NO_2_ and SO_2_ are associated with a significative interference on neurodevelopment by altering the brain structure [78,79]. For example, high prenatal PM_2.5_ exposure reduces cortical thickness, thinness of grey matter, and the corpus callosum volume; these slow down learning processes, impulse control, and the neurodevelopment of children [23,25,80]. NO_2_ exposure impairs the psychomotor development of children, the offspring of women who live in high-traffic areas are found to have reduced memory and attention functions due to NO_2_ exposure [81]. This is also supported by some of the studies identified in the present review. Prenatal exposure to PM_2.5_ and NO_2_ can lead to the development of ADHD symptoms later in children [52,54,55,56,61,62,64,65]. Other pollutants, such as PAHs and phthalates, are endocrine-disrupting chemicals known to, by reducing the white matter, alter the brain structure surface leading to impairment in brain functioning [82,83]. The same was observed for prenatal PAH exposure and the development of ADHD symptoms in the studies conducted by Perera et al. (2012, 2014 and 2018) [51,53,58] and Pagliaccio et al. (2022) [60], whereas a hypothetical mechanism suggests, from mice experimentation, that exposure to SO_2_ can increase the lipid peroxidation and produce reactive oxygen species, which can relocate to the central nervous system (CNS), causing inflammation in the neuro system, hereafter causing the development of ADHD symptoms [84]. Furthermore, SO_2_ and CO inhaled during the third trimester can affect the rapid development of neurons and neuronal interconnections in children’s brain [85]. Although only limited studies addressed the effects of prenatal CO, SO_2_, and O_3_, it has been suggested that the effect of being exposed to that pollutant has more impact during the third trimester of pregnancy [61,65].

A number of different studies explored a potential link between prenatal exposure to some air pollutants, such as NO_2_, PM, and PAH, and the development of ADHD in children. Most of the papers supporting this association were deemed to have a low risk of bias. However, some studies, such as Gong et al. 2014 [52], Pagliaccio et al. 2020 [60], and McGuinn et al. 2020 [61], found an association between these air pollutants and ADHD, but their risk of bias was moderate and their NOS quality was good. On the other hand, research on other pollutants, such as SO_2_, CO, and O_3_, is still in its early stages, but studies have shown a low risk of bias and good or very good NOS quality. Overall, 8 out of the 15 studies were judged to have a moderate risk of bias in outcome measurement. Therefore, while an association has been found between prenatal air pollutants and ADHD, the results must be interpreted with caution due to the risk of bias and methodological heterogeneity found between studies.

Consequently, future studies should aim to minimize heterogeneity and reduce bias by ensuring a more representative sample population, standardizing exposure, outcome assessments, and controlling the period of exposure during pregnancy. It is also recommended to use the WHO risk of bias assessment instrument [38] as a reference to advance research in this field.

### 4.1. Selected Studies Limitations

There were some limitations identified in the studies during the systematic review. The assessment of ADHD was different in most cases. The authors used various diagnostic criteria and a wide range of assessment tools for ADHD symptoms. Moreover, there was a lack of consideration of some important covariates, such as ADHD genetic risk and key prenatal risk factors. Additionally, the mode of collecting information on pollutant exposure methods varied from study to study, such as the land regression model, Gaussian dispersion model, station, blood samples, and personal data monitoring system.

### 4.2. Limitations and Strengths of the Review

One limitation of the current review was that the selected studies were different from one another, ranging from pollutant exposure measurement, ADHD symptoms assessment and diagnosis criteria, and sample size. Due to all these factors, it was not possible to carry out a further meta-analysis. One major strength of this systematic review is that it focused on only prenatal air pollutant exposure, and thoroughly followed PRISMA guidelines, which helped make the reporting data clear. The screening process and NOS quality and WHO risk of bias assessments were performed by 2–3 researchers independently. The selected articles were reported to be of good quality.

Due to the great heterogeneity found across study methodologies, such as pollutants and their measurement methods, covariates, ADHD assessment, diagnosis criteria and sample size, it becomes difficult to draw a firm conclusion. Although previous evidence explains the relation between air pollution and different neurodevelopment disorders, there is still a need for further exploration of the topic and its implication on children’s health.

## 5. Conclusions

In conclusion, the present systematic review provides summarized data on prenatal air pollutants exposure and its effect on the development of ADHD in children from 15 studies. Most of the studies reported an association between ADHD and prenatal PAH and PM exposure. However, data available on NO_2_ and SO_2_ were inconsistent, whereas the effect of CO/O_3_ is barely investigated by the researchers. Although epidemiological studies suggest that air pollutants can be contributing to ADHD, data on prenatal exposure need to be explored thoroughly due to the heterogeneity observed by the odd ratios forest plot, and the identified discrepancies in methodologies across the studies. Overall, eight of the fifteen studies were judged to be of moderate risk of bias in the outcome measurement. Due to these reasons, it is recommended that the results are interpreted with caution, and future studies should aim to minimize heterogeneity and reduce risk of bias by ensuring a more representative sample, standardizing exposure, and outcome assessments. More research needs to be conducted on this topic; specifically, each of the pollutants and methodologies should be considered and kept consistent to better understand the effect of air pollution exposure during pregnancy.

## Figures and Tables

**Table 1:** Epidemiological studies’ descriptions on prenatal/pregnancy air pollutants and ADHD/hyperactivity disorder.

Author/Year/Location/Design	Participants	Exposure Time	Exposure Measurement and Method	Pollutants	ADHD Symptoms and Diagnosis	Covariates	Results
**Perera et al. (2012)** USA Cohort [51]	253 children aged 6–7 years	Pregnancy	PAH measured by personal air monitoring of the mothers during pregnancy and DNA adducts specific to benzo[a]pyrene (BaP), a representative PAH, in maternal and cord blood.	PAH	Tool: CBCL/6–18. Method: The questionnaire was completed by mothers under the guidance of research workers trained in neurodevelopmental testing.	Child: sex, prenatal ETS. Mother: gestational age, maternal IQ, maternal education, ethnicity, prenatal demoralization, and age at assessment Family/environment: heating season and home caretaking environment (HOME inventory).	High prenatal PAH exposure was positively associated with symptoms in the CBCL syndromic scales of anxious/depressed and attention problems.
**Gong et al. (2014)** Sweden Cohort [52]	3426 twins’ parents. Children aged 9 or 12 years	Pregnancy, child’s first year	Air pollution concentrations at residential address during pregnancy of the mother and child‘s first year of life by dispersion model to obtain historical emission database on NO_x_ and PM (PM_10_).	NO_x_ and PM (PM_10_)	Tool: children neurodevelopment outcomes measures with A-TAC. Method: a telephone interview was conducted with parents.	Child: gender. Mother: gestational age, birth weight, maternal age at birth and maternal smoking during pregnancy. Family/environment: parity, neighbourhood deprivation index at child’s birth, and socio-economic data (maternal marital status, parental education, family income, and family size).	No clear association was found between air pollutants (NO_x_ and PM_10_) and during pregnancy and in the first of child’s life and ADHD.
**Perera et al. (2014)** USA Cohort [53]	233, children aged 9 years	Pregnancy	Prenatal PAH exposure estimated by levels of PAH-DNA adducts in maternal and cord blood collected at delivery.	PAH	Tool: CBCL/6–18 and CPRS–Revised: long version. Method: questionnaires were answered by mothers under the guidance of trained research worker.	Child: gender, child ethnicity, and prenatal ETS. Mother: gestational age, maternal intelligence, maternal education, maternal demoralization score, and maternal ADHD. Family/environment: heating season and home caretaking environment (HOME inventory).	High maternal adducts were positivity associated with the DSM-oriented attention deficit/hyperactivity problems scale on the CBCL, albeit not significant.
**Fuertes et al. (2016)** Germany Cohort [54]	4745 children aged 10 and 15 years	At birth/pregnancy	NO_2_, PM_10_ mass and absorbance assigned at child’s birth, 10- and 15 years home address using land-use regression model.	NO_2_ and PM (PM_10_, PM_2.5_ mass and absorbance	Tool: SDQ parent and self-reported versions. Method: the questionnaire was parent-informed at age of 10 years and self-informed at age of 15 years.	Child: sex. Mother: exact age at follow-up, parental education, maternal age at birth, smoking during pregnancy, second-hand smoke exposure, time spent in front of the screen, time spent outside, and single parent status. Family/environment: parental psychopathology.	Hyperactivity/inattention symptoms at 10 and 15 years were associated with PM_2.5_ mass and absorbance estimated.
**Yorifuji et al. (2016)** Japan Cohort [55]	33,911 children at 2.5 and 5.5 age	Pregnancy	Air pollutants measuring throughout stations in Japan database managed by the National Institute for Environmental Studies in Japan during pregnancy of mothers.	SPM, NO_2_ and SO_2_	Tool: national survey including three ADHD questions related to attention, self-regulation, and socially appropriate behaviours at the age of 5.5 years. Methods: items were completed by parents using “yes” or “no” categories.	Child: sex, birth month. Mother: maternal age, maternal education, and maternal smoking habit. Family/environment: parental income at the birth year of the child, type of residential area, per capita taxable income, population density of each municipality, and parity.	Air pollution exposure, in particular SPM, NO_2_ and SO_2_, during gestation were positively associated with risk for behavioural problems related to attention and delinquent or aggressive behaviour.
**Yorifuji et al. (2017)** Japan Cohort [56]	33,911 children at 8 years	Pregnancy	Measured at all general monitoring stations throughout Japan database managed by the National Institute for Environmental Studies in Japan during pregnancy of mothers.	SPM, NO_2_ and SO_2_	Tool: CBCL/4–18, Japanese edition and three questions related to attention problems: (1) Does your child interrupt people? (2) Can your child wait his/her turn during play? (3) Can your child pay attention to the surrounding area when crossing the street? Method: parent-informed questionnaire at child’s 8 years of age.	Child: sex, birth month. Mother: maternal age, maternal education, and maternal smoking habit. Family/environment: parental income at the birth year of the child, type of residential area, per capita taxable income, population density of each municipality, and parity.	Positive association between prenatal traffic-related air pollution exposure, in particular suspended PM, and behavioural developmental delays related to attention, self-regulation and socially appropriate behaviour was found at age 5.5 years. Additionally, observed decreased in self-inhibitory control and poor impulsive control; behaviours associated with ADHD.
**Forns et al. (2018)** European countries (Denmark, Netherlands, Germany, Italy, Spain, Sweden, and France) Cohort [57]	29,127 children aged 3-10 years	Pregnancy	Air pollutant concentrations measured at participants’ home addresses at birth and during pregnancy, using land-use regression model.	NO_2_ and PM (PM_10_, PM_2.5_, PM_coarse_ and absorbance	Tool: A-TAC, CBCL/1 ½–5, SDQ parent version and ADHD-DSM-IV list. Method: the questionnaires were completed by parents (A-TAC, CBCL 1 ½–5, SDQ) and by teachers (ADHD-DSM-IV scale).	Child: age, sex, and season of birth. Mother: level of education, age at delivery, country of birth, prenatal smoking, mother’s height, pre-pregnancy weight, body mass index. Family/environment: type of living area.	No association was found with air pollution during pregnancy and ADHD symptoms in children.
**Perera et al. (2018)** USA Cohort [58]	351 children aged 9 years	Pregnancy	PAH exposure, PAH-DNA adducts were measured in maternal blood at delivery.	PAH	Tool: CBCL/6–18, CPRS—Revised: long version. Method: reported by mothers and under the guidance of trained research worker.	Child: sex, child anxiety and depression symptoms at 9 years old, ethnicity, and nonverbal intelligence (TONI-2). Mother: ethnicity, prenatal ETS, maternal education, gestational age, age at assessment, and maternal ADHD. Family/environment: heating season and home caretaking environment (HOME inventory).	Children with high prenatal PAH exposure (adducts) developed more ADHD symptoms than those with low prenatal PAH exposure.
**Oudin et al. (2019)** Sweden Cohort [59]	>48,000 children	Pregnancy born between 1998–2006	Concentration of NO_x_ obtained from local sources, such as traffic using Gaussian dispersion model for NO_x_ (AERMOD).	NO_x_	Tool: None Method: Information about children with ADHD was extracted from public databases. ADHD diagnosis criteria: ICD-10.	Child: sex Mother: parental birth country, potential perinatal and maternal risk factors (maternal smoking, maternal age) Family/environment: socio-economic status (maternal education, family income), distance from psychiatric unit.	No associations were found between prenatal NO_x_ exposure and the risk of developing ADHD.
**Pagliaccio et al. (2020)** USA Cohort [60]	319 children	3rd trimester of Pregnancy born between 1998–2006	Personal air monitoring collected an external measure of exposure to PAH and maternal PAH-DNA adducts were also collected from a smaller subset.	PAH	Tool: CBCL/1½–5 and CBCL/6–18 years, CPRS-Revised, ADHD rating scale IV Method: Questionnaires were completed by parents under guidance of a trained research staff.	Child: sex Mother: gestational age, ethnicity, maternal IQ, maternal years of education. Family/environment: presence of a smoker in the home, quality of the proximal caretaking environment at age 3, and change of residence by age 5.	Children with higher prenatal PAH exposure showed significant association between postnatal ELS and CBCL attention and thought problems T-scores.
**McGuinn et al. (2020)** Mexico Cohort [61]	539 mother–child pairs	Pregnancy (2nd trimester)	1 km based satellite-based estimation model.	PM_2.5_	Tool: BASC-2 Method: questionnaires were completed by parents.	Child: sex, age. Mother: maternal age at enrolment, years of education, depressive symptoms during pregnancy, and maternal IQ. Family/environment: SES, home caretaking environment (HOME inventory), and season of conception.	An association between prenatal first trimester PM_2.5_ exposure and increases in the scores of several behavioural subscales, including attention problems and hyperactivity, was observed.
**Shih et al. (2020)** Taiwan Cohort [62]	16,376 mother–infant pairs. Children aged 8 years	Pregnancy	Air pollution data retrieved from fixed-site stations using nondispersive infrared spectroscopy for NO, NO_2_, NO_x_, CO, SO_2_; beta-ray attenuation for PM.	NO, NO_2_, NO_x_, SO_2_, PM (PM_10_ or less)	Tool: none Method: data extracted from Taiwan National Birth Registry from 2005. ADHD diagnosis criteria: not specified. Parents were asked for previous clinical diagnosis.	Child: sex, birth in summer (June–August). Mother: maternal age, delivery method. Family/environment: urban or rural residence, annual household income.	The occurrence of hyperactivity was significantly related to prenatal nitrogen oxide (NO_x_), but not to particulate matter 10 μm or less in diameter or SO_2_. Further analysis to separate effects by nitrogen dioxide (NO_2_) and/or nitric oxide (NO) showed that only NO was significantly related to hyperactivity.
**Peterson et al. (2022)** USA Cohort [63]	332 children aged 6–14 years	third trimester of pregnancy.	PM_2.5_ exposure measured for each day of pregnancy using spatiotemporal exposure models at home addresses; PAH exposure measured using personal air monitoring of the mothers over a 48 h period in the third trimester of pregnancy that collected vapours and particles ≥2.5 μg in diameter on a quartz microfiber filter.	PM_2.5_, PAH	Tool: ADHD severity Rating Scale. Method: children completed a detailed neuropsychological assessment at the time of MRI scanning, and mothers reported child’s social and emotional functioning.	Child: sex, child handedness. Mother: maternal ethnicity and maternal education. Family/environment: material hardship and quality of home environment.	Prenatal exposure to PAH and PM_2.5_ were found not significantly associated with any of the behavioural outcomes, ADHD severity or anxiety severity.
**Chang et al. (2022)** China Cohort [64]	425,736 children aged 5 years	Pregnancy	1 km based satellite-based estimation model.	PM_2.5_	Tool: none Method: data extracted from Taiwan National Birth Registry from 2004–2011. ADHD diagnosis criteria: ICD-9-CM.	Child: sex, birth weight, preterm birth, iron deficiency anaemia, asthma, atopic eczema, allergic rhinitis. Mother: maternal age at delivery, anaemia, heart disease, chronic diabetes, gestational diabetes mellitus, polyhydramnios and oligohydramnios, chronic hypertension, gestational hypertension, preeclampsia, maternal smoke, and drug use. Family/environment: SES.	The hazard ratio (HR) of ADHD was significantly associated with a 10 μg/m^3^ increase in PM_2.5_ during the first trimester and increased at PM_2.5_ over 16 μg/m^3^.
**Liu et al. (2022)** China Cohort [65]	26,052 children aged 3 years	Pregnancy	Air pollutants measured using land-use random forest (LURF) model collected from 57 monitoring stations.	SO_2_, NO_2_, CO, O_3_, PM_2.5_, PM_10_	Tool: CPRS-48 Method: questionnaires were completed by parents.	Child: sex, age, average daily sleep duration, feeding pattern, and birth weight. Mother: maternal age at birth, delivery way, parity, maternal passive smoking or alcohol consumption during pregnancy, multivitamins, folic acid or calcium supplementation during pregnancy, and gestational diseases. Family/environment: paternal education and age at birth, family income, parent–child interactive activities, number of persons in house, prenatal household air pollution conditions (fumes from cooking, ETS, home renovation, mosquito coils, and burning of incense indoors), ETS, and monthly mean ambient temperature during first three years of age.	An association was found between air pollutants NO_2_, PM_10_, PM_2.5_ and hyperactivity. Moreover, the risk of hyperactivity significantly increased with 10 μg/m^3^ exposure from 7th month of pregnancy to 4th month after birth and peak was noticed at 9th month of pregnancy. However, no significant relation observed for air pollutants CO, O_3_, and SO_2_ and child hyperactivity.

Abbreviations: SPM (suspended particulate matter), PM (particulate matter), NO (nitric oxide), NO_x_ (nitrogen oxide), NO_2_ (nitrogen dioxide), SO_2_ (sulphur dioxide), O_3_ (ozone), PAH (polycyclic aromatic hydrocarbons), A-TAC (The Autism–Tics, ADHD and other Comorbidities inventory), CBCL (Child Behaviour Checklist) for 1 ½–5 years of age; 6–18 years of age; and 4–18 years of age, SDQ (Strengths and Difficulties Questionnaire), DSM (Diagnostic and Statistical Manual of Mental Disorders), ADHD-DSM-IV (ADHD Criteria of Diagnostic and Statistical Manual of Mental Disorders, Fourth Edition), CPRS (Conners Parent Report Scale), ICD-9 or 10 (International Classification of Mental and Behavioural Disorders Version 9 or 10); BASC-2 (Behavioural Assessment System for Children, second edition); TONI (Test of Non-Verbal Intelligence), HOME (Home Observation for Measurement of the Environment), ELS (early life stress); SES (socio economic status).

**Table 2:** Newcastle–Ottawa scale (NOS) quality assessment.

Paper	Study Design	Selection (Max 4 Stars)	Comparability (Max 2 Stars)	Outcome (Max 3 Stars)	Total Score
[51]	Cohort	★ ★ ★ ★	★ ★	★	7/9
[52]	Cohort	★ ★ ★	★ ★	★ ★	7/9
[53]	Cohort	★ ★ ★ ★	★ ★	★	7/9
[54]	Cohort	★ ★ ★ ★	★ ★	★ ★	8/9
[55]	Cohort	★ ★ ★ ★	★ ★	★	7/9
[56]	Cohort	★ ★ ★ ★	★ ★	★	7/9
[57]	Cohort	★ ★ ★ ★	★ ★	★ ★	8/9
[58]	Cohort	★ ★ ★ ★	★ ★	★	7/9
[59]	Cohort	★ ★ ★ ★	★ ★	★ ★ ★	9/9
[60]	Cohort	★ ★ ★ ★	★ ★	★	7/9
[61]	Cohort	★ ★ ★	★ ★	★	6/9
[62]	Cohort	★ ★ ★ ★	★★	★ ★★	9/9
[63]	Cohort	★ ★ ★ ★	★	★ ★	7/9
[64]	Cohort	★ ★ ★ ★	★★	★ ★★	9/9
[65]	Cohort	★ ★ ★ ★	★★	★	7/9

The NOS total score ranges between zero up to nine stars to indicate the quality of the study: 9–8 (very good), 7–6 (good), 5–4 (satisfactory), and 3–0 (unsatisfactory).

**Table 3:** Risk of bias assessment heat map.

	Assessment Domain					
Paper	Confounding	Selection Bias	Exposure Assessment	Outcome Measurement	Missing Data	Selective Reporting
[51]						
[52]						
[53]						
[54]						
[55]						
[56]						
[57]						
[58]						
[59]						
[60]						
[61]						
[62]						
[63]						
[64]						
[65]						
						
						
						
		**Risk of Bias**				
						

green = Low; yellow = Moderate; orange = High.

## Data Availability

The data presented in this study are available within the article and Supplementary Material.

## Supplementary Materials

The following supporting information can be downloaded at: https://www.mdpi.com/article/10.3390/ijerph20085443/s1, Table S1: PECOS criteria for inclusion and exclusion of studies; Table S2: Newcastle-Ottawa Scale assessment and World Health Organization Risk of Bias Assessment.
