# Supplementary material for: Prenatal Exposure to Air Pollutants and Attentional Deficit Hyperactivity Disorder Development in Children: A Systematic Review
Source: Int J Environ Res Public Health. 2023 Apr 7;20(8):5443. doi: 10.3390/ijerph20085443 (PMC10138804; doi:10.3390/ijerph20085443)
Supplement: Supplementary file 1 [file ijerph-20-05443-s001.zip › ijerph-2208396-supplementary.pdf]

PRENATAL EXPOSURE TO AIR POLLUTANTS AND ATTENTIONAL DEFICIT HYPERACTIVITY DISORDER DEVELOPMENT IN CHILDREN: A SYSTEMATIC REVIEW

Supplementary Table S1: PECOS criteria for inclusion and exclusion of studies

Detailed PECOS (population, exposure, comparison, outcome, study type) table for the systematic review on air pollutants and ADHD.

	Population	Exposure	Comparison	Outcome	Type of study
**Included**	Pregnant women/Child population	Air pollutants PM, NO_x, SO_2, O_3, PAH	Not applicable	ADHD/hyperactivity	Observational studies (cohort, case-control and cross sectional)
**Excluded**	Adults, in vitro and animal studies	Any other pollutants (e.g, bisphenol A)	Not applicable	Other neurodevelopmental disorder (e.g. autism)	Controlled trials<br>Letters to the editor<br>Reviews<br>Case report<br>Interventional studies<br>Abstracts

Supplementary Table S2: Newcastle-Ottawa Scale (NOS) quality and World Health Organization **(**WHO) Risk of Bias Assessment

**1. Perera et al., 2012**		
Design	Cohort study	
Participants	Children, aged 6-7 years	
Exposure	PAH	
Outcomes	Attention-Deficit/Hyperactivity Disorder	
**Quality Assessment**		
**Newcastle-Ottawa Quality Assessment Scale-Case Cohort Study**		**Author's judgement**
**Selection**	Representative of the exposed cohort	*
	Selection of the non-exposed cohort	*
	Ascertainment of exposure	*
	Demonstration that outcome of interest was not present at start of study	*
**Comparability**	Comparability of cohorts on the basis of the design of analysis	**
**Outcome**	Assessment of outcome	
	Was follow-up long enough for outcome to occur	*
	Adequate of follow up of cohorts	

**World Health Organization - Risk of Bias Assessment**	
**Domain**	**Author's judgement**
Confounding	Low
Selection Bias	Low
Exposure Assessment	Low
Outcome measurement	Low
Missing data	Low
Selective reporting	Low

**2. Gong et al., 2014**		
Design	Cohort study	
Participants	Children, aged 9-12 years	
Exposure	PM_{10}, NO_x	
Outcomes	Attention-Deficit/Hyperactivity Disorder	
**Quality Assessment**		
**Newcastle-Ottawa Quality Assessment Scale-Case Cohort Study**		**Author's judgement**
**Selection**	Representative of the exposed cohort	
	Selection of the non-exposed cohort	*
	Ascertainment of exposure	*
	Demonstration that outcome of interest was not present at start of study	*
**Comparability**	Comparability of cohorts on the basis of the design of analysis	**
**Outcome**	Assessment of outcome	*
	Was follow-up long enough for outcome to occur	*
	Adequate of follow up of cohorts	

**World Health Organization - Risk of Bias Assessment**	
**Domain**	**Author's judgement**
Confounding	Low
Selection Bias	Low
Exposure Assessment	Moderate
Outcome measurement	Moderate
Missing data	Moderate
Selective reporting	Low

3. Perera et al., 2014		
Design	Cohort study	
Participants	Children, aged 9 years	
Exposure	PAH	
Outcomes	Attention-Deficit/Hyperactivity Disorder	
**Quality Assessment**		
**Newcastle-Ottawa Quality Assessment Scale-Case Cohort Study**		**Author's judgement**
**Selection**	Representative of the exposed cohort	*
	Selection of the non-exposed cohort	*
	Ascertainment of exposure	*
	Demonstration that outcome of interest was not present at start of study	*
**Comparability**	Comparability of cohorts on the basis of the design of analysis	**
**Outcome**	Assessment of outcome	
	Was follow-up long enough for outcome to occur	*
	Adequate of follow up of cohorts	

World Health Organization - Risk of Bias Assessment	
**Domain**	**Author's judgement**
Confounding	Low
Selection Bias	Low
Exposure Assessment	Low
Outcome measurement	Low
Missing data	Low
Selective reporting	Low

4. Fuertes et al., 2016		
Design	Cohort study	
Participants	Children, aged 10-15 years	
Exposure	PM_{10}, $PM_{2.5\ mass}$, $PM_{2.5\ absorbance}$ and NO_2	
Outcomes	Attention-Deficit/Hyperactivity Disorder	
**Quality Assessment**		
**Newcastle-Ottawa Quality Assessment Scale-Case Cohort Study**		**Author's judgement**
**Selection**	Representative of the exposed cohort	*
	Selection of the non-exposed cohort	*
	Ascertainment of exposure	*
	Demonstration that outcome of interest was not present at start of study	*
**Comparability**	Comparability of cohorts on the basis of the design of analysis	**
**Outcome**	Assessment of outcome	
	Was follow-up long enough for outcome to occur	*
	Adequate of follow up of cohorts	*

**World Health Organization - Risk of Bias Assessment**	
**Domain**	**Author's judgement**
Confounding	Low
Selection Bias	Low
Exposure Assessment	Low
Outcome measurement	Moderate
Missing data	Moderate
Selective reporting	Low

5. Yorifuji et al., 2016		
Design	Cohort study	
Participants	Children, aged 2.5 - 5.5 years	
Exposure	SPM, NO_2, SO_2	
Outcomes	Attention-Deficit/Hyperactivity Disorder	
**Quality Assessment**		
**Newcastle-Ottawa Quality Assessment Scale-Case Cohort Study**		**Author's judgement**
**Selection**	Representative of the exposed cohort	*
	Selection of the non-exposed cohort	*
	Ascertainment of exposure	*
	Demonstration that outcome of interest was not present at start of study	*
**Comparability**	Comparability of cohorts on the basis of the design of analysis	**
**Outcome**	Assessment of outcome	
	Was follow-up long enough for outcome to occur	*
	Adequate of follow up of cohorts	
**World Health Organization - Risk of Bias Assessment**		
**Domain**	**Author's judgement**	
Confounding	Low	
Selection Bias	Low	
Exposure Assessment	Low	
Outcome measurement	Moderate	
Missing data	Low	
Selective reporting	Low	

6. Yorifuji et al., 2017		
Design	Cohort study	
Participants	Children, aged 8 years	
Exposure	SPM, NO_2, SO_2	
Outcomes	Attention-Deficit/Hyperactivity Disorder	
**Quality Assessment**		
**Newcastle-Ottawa Quality Assessment Scale-Case Cohort Study**		**Author's judgement**
**Selection**	Representative of the exposed cohort	*
	Selection of the non-exposed cohort	*
	Ascertainment of exposure	*
	Demonstration that outcome of interest was not present at start of study	*
**Comparability**	Comparability of cohorts on the basis of the design of analysis	**
**Outcome**	Assessment of outcome	
	Was follow-up long enough for outcome to occur	*
	Adequate of follow up of cohorts	

**World Health Organization - Risk of Bias Assessment**	
**Domain**	**Author's judgement**
Confounding	Low
Selection Bias	Low
Exposure Assessment	Low
Outcome measurement	Low
Missing data	Low
Selective reporting	Low

7. Forns et al., 2018		
Design	Cohort study	
Participants	Children, aged 3-10 years	
Exposure	PM_{10}, $PM_{2.5}$, PM_{coarse} and $PM_{absorbance}$	
Outcomes	Attention-Deficit/Hyperactivity Disorder	
**Quality Assessment**		
**Newcastle-Ottawa Quality Assessment Scale-Case Cohort Study**		**Author's judgement**
**Selection**	Representative of the exposed cohort	*
	Selection of the non-exposed cohort	*
	Ascertainment of exposure	*
	Demonstration that outcome of interest was not present at start of study	*
**Comparability**	Comparability of cohorts on the basis of the design of analysis	**
**Outcome**	Assessment of outcome	
	Was follow-up long enough for outcome to occur	*
	Adequate of follow up of cohorts	*

**World Health Organization - Risk of Bias Assessment**	
**Domain**	**Author's judgement**
Confounding	Low
Selection Bias	Low
Exposure Assessment	Low
Outcome measurement	Moderate
Missing data	Moderate
Selective reporting	Low

8. Perera et al., 2018			
Design	Cohort study		
Participants	Children, aged 9 years		
Exposure	PAH		
Outcomes	Attention-Deficit/Hyperactivity Disorder		
**Quality Assessment**			
**Newcastle-Ottawa Quality Assessment Scale-Case Cohort Study**			**Author's judgement**
**Selection**	Representative of the exposed cohort		*
	Selection of the non-exposed cohort		*
	Ascertainment of exposure		*
	Demonstration that outcome of interest was not present at start of study		*
**Comparability**	Comparability of cohorts on the basis of the design of analysis		**
**Outcome**	Assessment of outcome		
	Was follow-up long enough for outcome to occur		*
	Adequate of follow up of cohorts		

World Health Organization - Risk of Bias Assessment	
**Domain**	**Author's judgement**
Confounding	Low
Selection Bias	Low
Exposure Assessment	Low
Outcome measurement	Low
Missing data	Low
Selective reporting	Low

9. Oudin et al., 2019	
Design	Cohort study
Participants	Children born between 1999 - 2009
Exposure	NO_x
Outcomes	Attention-Deficit/Hyperactivity Disorder
**Quality Assessment**	

**Newcastle-Ottawa Quality Assessment Scale-Case Cohort Study**		**Author's judgement**
**Selection**	Representative of the exposed cohort	*
	Selection of the non-exposed cohort	*
	Ascertainment of exposure	*
	Demonstration that outcome of interest was not present at start of study	*
**Comparability**	Comparability of cohorts on the basis of the design of analysis	**
**Outcome**	Assessment of outcome	*
	Was follow-up long enough for outcome to occur	*
	Adequate of follow up of cohorts	*

**World Health Organization - Risk of Bias Assessment**	
**Domain**	**Author's judgement**
Confounding	Low
Selection Bias	Low
Exposure Assessment	Moderate
Outcome measurement	Low
Missing data	Low
Selective reporting	Low

10. Pagliaccio et al., 2020		
Design	Cohort study	
Participants	Children born between 1998 - 2006	
Exposure	PAH	
Outcomes	Attention-Deficit/Hyperactivity Disorder	
**Quality Assessment**		
**Newcastle-Ottawa Quality Assessment Scale-Case Cohort Study**		**Author's judgement**
**Selection**	Representative of the exposed cohort	*
	Selection of the non-exposed cohort	*
	Ascertainment of exposure	*
	Demonstration that outcome of interest was not present at start of study	*
**Comparability**	Comparability of cohorts on the basis of the design of analysis	**
**Outcome**	Assessment of outcome	
	Was follow-up long enough for outcome to occur	*
	Adequate of follow up of cohorts	

World Health Organization - Risk of Bias Assessment	
**Domain**	**Author's judgement**
Confounding	Moderate
Selection Bias	Moderate
Exposure Assessment	Low
Outcome measurement	Moderate
Missing data	High
Selective reporting	Low

11. McGuinn et al., 2020			
Design	Cohort study		
Participants	Children – mother pair		
Exposure	$PM_{2.5}$		
Outcomes	Attention-Deficit/Hyperactivity Disorder		
**Quality Assessment**			
**Newcastle-Ottawa Quality Assessment Scale-Case Cohort Study**			**Author's judgement**
**Selection**	Representative of the exposed cohort		*
	Selection of the non-exposed cohort		
	Ascertainment of exposure		*
	Demonstration that outcome of interest was not present at start of study		*
**Comparability**	Comparability of cohorts on the basis of the design of analysis		**
**Outcome**	Assessment of outcome		
	Was follow-up long enough for outcome to occur		*
	Adequate of follow up of cohorts		

World Health Organization - Risk of Bias Assessment	
**Domain**	**Author's judgement**
Confounding	Low
Selection Bias	Moderate
Exposure Assessment	Moderate
Outcome measurement	Low
Missing data	High
Selective reporting	Low

12. Shih et al., 2020		
Design	Cohort study	
Participants	Children, aged 8 years	
Exposure	NO_x, NO_2, SO_2, $PM_{10 \text{ or less}}$	
Outcomes	Attention-Deficit/Hyperactivity Disorder	
**Quality Assessment**		
**Newcastle-Ottawa Quality Assessment Scale-Case Cohort Study**		**Author's judgement**
**Selection**	Representative of the exposed cohort	*
	Selection of the non-exposed cohort	*
	Ascertainment of exposure	*
	Demonstration that outcome of interest was not present at start of study	*
**Comparability**	Comparability of cohorts on the basis of the design of analysis	**
**Outcome**	Assessment of outcome	*
	Was follow-up long enough for outcome to occur	*
	Adequate of follow up of cohorts	*

**World Health Organization - Risk of Bias Assessment**	
**Domain**	**Author's judgement**
Confounding	Low
Selection Bias	Low
Exposure Assessment	Low
Outcome measurement	High
Missing data	Moderate
Selective reporting	Low

13. Peterson et al., 2022		
Design	Cohort study	
Participants	Children, aged 6-14 years	
Exposure	$PM_{2.5}$, PAH	
Outcomes	Attention-Deficit/Hyperactivity Disorder	
**Quality Assessment**		
**Newcastle-Ottawa Quality Assessment Scale-Case Cohort Study**		**Author's judgement**
**Selection**	Representative of the exposed cohort	*
	Selection of the non-exposed cohort	*
	Ascertainment of exposure	*
	Demonstration that outcome of interest was not present at start of study	*
**Comparability**	Comparability of cohorts on the basis of the design of analysis	*
**Outcome**	Assessment of outcome	*
	Was follow-up long enough for outcome to occur	*
	Adequate of follow up of cohorts	

World Health Organization - Risk of Bias Assessment	
**Domain**	**Author's judgement**
Confounding	Low
Selection Bias	Low
Exposure Assessment	Moderate
Outcome measurement	Low
Missing data	Low
Selective reporting	Low

14. Chang et al., 2022			
Design	Cohort study		
Participants	Children, aged 5 years		
Exposure	$PM_{2.5}$		
Outcomes	Attention-Deficit/Hyperactivity Disorder		
**Quality Assessment**			
**Newcastle-Ottawa Quality Assessment Scale-Case Cohort Study**			**Author's judgement**
**Selection**	Representative of the exposed cohort		*
	Selection of the non-exposed cohort		*
	Ascertainment of exposure		*
	Demonstration that outcome of interest was not present at start of study		*
**Comparability**	Comparability of cohorts on the basis of the design of analysis		**
**Outcome**	Assessment of outcome		*
	Was follow-up long enough for outcome to occur		*
	Adequate of follow up of cohorts		*

**World Health Organization - Risk of Bias Assessment**	
**Domain**	**Author's judgement**
Confounding	Low
Selection Bias	Low
Exposure Assessment	Low
Outcome measurement	Moderate
Missing data	Low
Selective reporting	Low

15. Liu et al., 2022		
Design	Cohort study	
Participants	Pregnancy	
Exposure	PM_{10}, $PM_{2.5}$, SO_2, NO_2, O_3, CO	
Outcomes	Attention-Deficit/Hyperactivity Disorder	
**Quality Assessment**		
**Newcastle-Ottawa Quality Assessment Scale-Case Cohort Study**		**Author's judgement**
**Selection**	Representative of the exposed cohort	*
	Selection of the non-exposed cohort	*
	Ascertainment of exposure	*
	Demonstration that outcome of interest was not present at start of study	*
**Comparability**	Comparability of cohorts on the basis of the design of analysis	**
**Outcome**	Assessment of outcome	
	Was follow-up long enough for outcome to occur	
	Adequate of follow up of cohorts	*

World Health Organization - Risk of Bias Assessment	
**Domain**	**Author's judgement**
Confounding	Low
Selection Bias	Low
Exposure Assessment	Low
Outcome measurement	Moderate
Missing data	Low
Selective reporting	Low
